# Unacceptable Experiences Reported by Undergraduate Students and Their Associations With Mental Health, Well-Being and Academic Performance: U-Flourish Student Well-Being Research: Expériences inacceptables signalées par les étudiants de premier cycle et leurs liens avec la santé mentale, le bien-être et le rendement académique : Programme de recherche U-Flourish sur le bien-être des étudiants

**DOI:** 10.1177/07067437251412566

**Published:** 2026-02-10

**Authors:** S. Hayden John, Anne Duffy, Alice Y.S. Li, Kristen Kyone, Emily Dephoure, Daniel Rivera, Adeleine Lyon, Nathan King

**Affiliations:** 1 Faculty of Health Sciences, 12363 Queen's University, Kingston, ON, Canada; 2 Department of Psychiatry, 4257 Queen's University, Kingston, ON, Canada; 3 Department of Psychiatry, 9830 Oxford University, Oxford, UK; 4 Department of Public Health Sciences, Queen's University, Kingston, ON, Canada; 5 School of Medicine, 4257 Queen's University, Kingston, ON, Canada; 6 Centre for Neuroscience Studies, Queen's University, Kingston, ON, Canada

**Keywords:** bullying, discrimination, educational measurement, harassment, mental health, sexual violence, students, unacceptable experiences, well-Being

## Abstract

**Objectives:**

Unacceptable experiences (UEs) during undergraduate studies and the associated emotional and academic impact have not been rigorously evaluated in the Canadian context. This study aimed to estimate the prevalence of UEs and examine their associations with mental health and academic outcomes in a diverse sample of Canadian undergraduate students.

**Methods:**

Undergraduates attending Queen's University completed the U-Flourish Student Well-Being Survey at the beginning and end of each academic year from 2021/2022 to 2023/2024. Validated self-report measures included the GAD-7 (anxiety), PHQ-9 (depression), C-SSRS (suicidal thoughts and behaviours) and WEMWBS-7 (well-being). UEs reported over the academic year included: discrimination, sexual violence/harassment, bullying, hate crimes and physical assault. Multivariable regression analyses examined associations between UEs and student mental health outcomes and cumulative grade-point average (GPA) abstracted from the university database.

**Results:**

Nearly one-third (28.9%; range = 27.0–30.1% across years) of students (*n = *2,948) reported experiencing at least one UE over the academic year. Discrimination (14.6%) and sexual violence/harassment (14.4%) was reported most frequently, followed by bullying/harassment (10.6%), hate crimes (5.0%) and physical assault (3.2%). UEs were highest in students who identified as non-binary gender (51.6%), 2SLGBTQIA + (39.9%) and as having a lifetime mental illness (41.2%). Each UE increased the risk of screening positive for anxiety and depression over the academic year by 10–19% and 14–40%, respectively. Students reporting UEs were also more likely (13–72%) to report having suicidal thoughts and/or behaviours over the academic year, particularly those reporting sexual violence (RR:1.72; 95% CI:1.45–2.05). Sexual violence, bullying, and hate crimes were associated with lower average cumulative GPA in first year.

**Conclusion:**

UEs were commonly reported by undergraduate students, especially in minoritised subgroups, and associated with mental health concerns and academic difficulties. These findings highlight the need for further research to inform universal and targeted prevention and early intervention initiatives.

## RÉSUMÉ

**Objectifs:**

Les expériences inacceptables (EI) vécues pendant les études de premier cycle et leurs répercussions émotionnelles et sur le rendement académique n'ont pas fait l'objet d'une évaluation rigoureuse dans le contexte canadien. L'étude visait à estimer la prévalence des EI et à examiner les liens entre la santé mentale et les résultats académiques dans un échantillon diversifié d'étudiants canadiens de premier cycle.

**Méthodes:**

Les étudiants de premier cycle de l'Université Queen's ont répondu au sondage U-Flourish sur le bien-être des étudiants au début et à la fin de chaque année universitaire de 2021-2022 à 2023-2024. Les mesures d'auto-évaluation validées comprenaient les questionnaires GAD-7 (anxiété), PHQ-9 (dépression), C-SSRS (pensées et comportements suicidaires) et WEMWBS-7 (bien-être). Les EI signalées au cours de l'année universitaire comprenaient la discrimination, la violence ou le harcèlement sexuels, l'intimidation, les crimes haineux et les agressions physiques. Des analyses de régression multivariables ont examiné les liens entre les EI et les résultats en matière de santé mentale des étudiants et la moyenne pondérée cumulative (MPC) extraite de la base de données universitaire.

**Résultats:**

Près du tiers (28,9 %; 27,0 à 30,1 %; 2021-2022 à 2023-2024) des étudiants (n = 2 948) ont déclaré avoir vécu au moins une EI au cours de l'année universitaire. La discrimination (14,6 %) ainsi que la violence et le harcèlement sexuels (14,4 %) ont été signalés le plus souvent, suivis de l'intimidation et du harcèlement (10,6 %), des crimes haineux (5,0 %) et des agressions physiques (3,2 %). La fréquence des EI était la plus élevée chez les étudiants qui se sont dits non binaires (51,6 %), 2SLGBTQIA+ (39,9 %) et ceux ayant été atteints d'un trouble de santé mentale au cours de leur vie (41,2 %). Chaque EI a augmenté le risque de dépistage positif pour l'anxiété et la dépression au cours de l'année universitaire de 10 à 19 % et de 14 à 40 %, respectivement. Les étudiants qui ont signalé des EI étaient également plus susceptibles (de 13 à 72 %) de signaler avoir eu des pensées ou des comportements suicidaires au cours de l'année universitaire, en particulier ceux qui avaient déclaré avoir été victimes de violence sexuelle (RR : 1,72; IC à 95 % : 1,45 à 2,05). La violence sexuelle, l'intimidation et les crimes haineux étaient associés à une MPC inférieure au cours de la première année.

**Conclusion:**

Les EI ont été fréquemment signalées par les étudiants de premier cycle, en particulier dans les sous-groupes minoritaires, et ont été associées à des troubles de santé mentale et à des difficultés académiques. Ces résultats soulignent la nécessité de mener d'autres recherches afin d'orienter les initiatives universelles et ciblées en matière de prévention et d'intervention précoce.

## Introduction

Unacceptable experiences (UEs) such as bullying, sexual violence, discrimination, and harassment during adolescence have been associated with significant social, psychological and mental health consequences.^1^ Few large-scale studies have estimated the prevalence of UEs among university students in Canada and more globally. However, from available evidence UEs among university students are common (i.e., ≥17%) with no indication of reduction despite emphasis on the importance of equity, diversity and inclusivity on university campuses.^2^ The compassionate campus movement has gained traction in parallel to efforts to facilitate historically disadvantaged young people in attending higher education.^3^ However, there is a major knowledge gap as to the impact of the compassionate campus movement on reported UEs.

In one Canadian study, approximately 45% of women and 32% of men reported experiencing sexual violence whilst attending postsecondary education in 2019.^4^ Hate crimes, defined as criminal acts committed against individuals based on race, religion, sexual orientation, sex, gender, or disability,^5^ differ from discrimination, which refers to unfair and prejudicial treatment of individuals without criminal intent.^6^ A 2011 study at two Canadian Universities found that 40% of students reported experiencing a hate crime or discrimination, most commonly in the form of verbal assault.^7^ Hate crimes can heighten feelings of social isolation and vulnerability, leading to chronic stress and long-term psychological distress.^8^ Studies have shown that students who feel safe at university perform better academically and are less susceptible to anxiety and depression, which are common correlates of unresolved trauma.^8^

Bullying, defined as unwanted aggressive behaviour involving a power imbalance, can take physical, verbal, or more covert forms.^9^ Although data on bullying specifically among university students is limited, a 2019 survey of Canadian youth aged 12–17 years showed that 42% reported experiencing some form of bullying at least monthly in the past year.^10,11^ Evidence consistently shows negative consequences of bullying on self-esteem, academic performance, and emotional and physical well-being.^12–14^ The prevalence of physical assault, defined as an act of threatening or committing physical harm towards someone,^15^ has reportedly increased among university students in recent years (2014–2019) and is associated with an increased risk of depression, anxiety, suicidal ideation and risk-taking, including substance abuse.^16^

Research on UEs among college and university students remains limited, with few studies examining their prevalence or their mental health and academic impacts, particularly among Canadian students, and most relying on cross-sectional designs. Given that most high school graduates attend higher education, come from increasingly diverse socio-cultural backgrounds and report increasing rates of anxiety, depression and associated self-harm,^17^ it is important to better understand the prevalence and impact of UEs on student mental health and academic performance. The objectives of this study were therefore to: (i) estimate the prevalence of UEs reported by a large diverse sample of undergraduate students attending a major Canadian university, and (ii) examine the associations between UEs and mental health, well-being and academic performance. To our knowledge, this study represents the first large-scale survey in Canada to examine both the prevalence of UEs and their impact on university students’ mental health and academic success.

## Methods

### Data Source

Data derived from the U-Flourish Well-being Survey study are described in detail elsewhere.^18^ Briefly, since fall 2018, all first-year undergraduate students entering Queen's University, Canada have been invited to complete an electronic U-Flourish Survey after reading a Letter of Information and providing electronic consent. After cross-campus student-led awareness and engagement campaigns, students are invited to complete the survey via their university email near the beginning (fall) and end (spring) of their first and subsequent academic years. The fall baseline survey collects demographic, familial, lifestyle, psychological, and mental health and well-being data. The spring survey collects follow-up data on mental health and well-being, UEs, and help-seeking during the academic year.

This study analysed data from students across all years of study who completed a spring survey during the 2021/2022 to 2023/2024 academic years, when UEs were measured. Approximately 5–8.5% of the undergraduate student population and 14–35% of first-year students completed the fall survey each year. Response rates on the spring follow-up surveys ranged from 43–49% across years.

The U-Flourish Survey study was reviewed for ethical compliance by the Queen's University and Affiliated Teaching Hospitals Research Ethics Board (HSREB PSIY-609-18).

### Measures

#### Demographic Characteristics

At baseline, students reported their age (years), gender [male, female, or non-binary (non-binary, do not self-identify with a label, or prefer not to say)], sexual orientation (heterosexual or 2SLGBTQIA+) and international student status. Ethnicity was identified through a standard list,^19^ and collapsed into: White, Black, Middle Eastern, South Asian, East or Southeast Asian and other (including students who selected multiple). Students reported the highest level of education completed by either parent (graduate degree, bachelor's degree/apprenticeship, or high school or less) and their lifetime history of diagnosed mental illness. Students’ program of study was obtained by linking to administrative university data.

#### Unacceptable Experiences (UEs)

Adapted from the Bristol University Student Wellbeing Survey^2^ unacceptable behaviour items, students were asked if they had experienced each of the following UEs (“*yes*”, “*no*”, or “*not sure*”) while studying at university during the past year: sexual violence or harassment, physical assault, bullying/harassment, hate crimes, or discrimination. “*Yes*” and “*not sure*” responses were combined for analysis, as they had similar associations with the mental health outcomes of interest (i.e., effects were largely in the same direction and similar in magnitude; see Supplementary Tables 1A, 1B & 2). Further, “*not sure*” responses were interpreted as a participant having had an unacceptable experience but being unsure whether it qualified. This rationale has been supported in recent mental health outcome-based studies.^20^ The proportion of students responding “*not sure*” ranged from 6.4% for discrimination and 4.6% for sexual violence or harassment to 1.3% for physical assault.

#### Mental Health Outcomes

Anxiety symptoms over the past 2 weeks were assessed using the 7-item Generalised Anxiety Disorder (GAD-7) scale (α= .92). Responses to the items (0=*“not at all”* to 3=*“nearly every day”*) were summed (range = 0–21), with higher scores indicating greater symptom severity.^21^ Depressive symptoms were similarly measured using the 9-item Patient Health Questionnaire (PHQ-9) (range = 0–27; α= .89).^22^ On both scales a score of ≥10 indicates a screen positive for clinically significant symptoms.^21,22^

Suicidal ideation and attempts during the academic year were assessed using the following items adapted from the Columbia Suicide Severity Rating Scale: “*Have you had thoughts about ending your life?”* and “*Have you made a suicide attempt?*”.^23^ Suicidal thoughts and behaviours were indicated by a “*Yes*” response to either question.

Well-being was measured using the Short Warwick-Edinburgh Mental Wellbeing Scale (WEMWBS-7),^24^ which includes seven items measuring psychological functioning and emotional states, such as “*I’ve been feeling useful*” (1=“*none of the time*” to 5=“*all of the time*”). Items were summed (α= .87), with cumulative scores of ≤19 indicative of “low well-being”.^25^

#### Academic Performance

Average cumulative Grade Point Average (GPA) over the academic year (Fall and Winter terms) was obtained by linking survey responses to Queen's administrative data, for all students except those in the medical program, which uses a pass/fail grading system. Queen's uses a GPA scale of 0 to 4.3, with 0 corresponding to a 0–49.9% (F) average and 4.3 corresponding to a 90–100% (A+) average (https://www.queensu.ca/registrar/academic-info/grades/official-gpa-scale).

#### Help-Seeking

Help-seeking (“*yes*” or “*no*”) was measured using the question: “*During this academic year, have you accessed any university wellness, counselling, mental health or learning support services?”.*

### Statistical Analysis

Analyses were conducted using SAS Version 9.4.^26^ Students excluded from the analysis due to missing data on the UE items (771/3,719 [20.7%]) were younger on average (19.2 [SD = 1.8] vs. 19.4 [SD = 1.9], *P* = .002), but similar by gender, minority ethnicity, program of study, and parental education level (*P* > .05). Spearman's correlation coefficients were used to assess the strength of association between UEs; a correlation of <0.4 was considered weak to negligible, 0.4–0.6 moderately strong and ≥0.7 strong.^27^ Descriptive statistics summarised the sample and UEs, overall, and by student demographic subgroups; 95% confidence intervals were adjusted to account for repeated measures. Rao-Scott chi-square tests,^28^ were used to examine whether the distribution of UEs differed by demographics, accounting for the non-independence of responses from students who completed the survey in multiple academic years (*n = *596).

Multivariable log-binomial regression was used to estimate Relative Risks (RRs) examining associations between each of the five UEs and mental health outcomes reported at follow-up. Generalised Estimating Equations (GEE) with unstructured working correlation structure were used to account for repeated measures from students who completed the survey in multiple years, by estimating and adjusting for within-subject correlation.^29^ All models adjusted for age, gender, lifetime mental illness, parental education and ethnicity, which were correlated with reporting a UE in our sample and have been previously linked to mental health.^2^ A total of 8.6% of students were excluded from the regression analyses due to missing covariate data. Multiple imputation was conducted as a sensitivity analysis to assess potential bias due to missing covariate data. The Markov Chain Monte Carlo method was used to generate 10 imputed datasets, incorporating all study variables.^30^

Models were run with and without adjustment for baseline mental health status to examine whether (i) students who reported a UE were more likely to report mental health problems, and (ii) whether UEs were associated with developing new problems or declining mental health over the academic year. This approach was applied to all mental health outcomes except suicidal thoughts and behaviours because baseline status was not consistently measured and therefore unavailable for the majority (65%) of the sample.

Multivariable linear regression was conducted to examine associations between UEs and cumulative GPA, adjusting for the same set of covariates and program of study. The associations were examined in the full sample, and in first-year students who are particularly vulnerable to mental health problems and academic struggles.^31^ Undergraduate medical students (*n = *111) were excluded from GPA analyses because they are graded on a pass/fail basis.

## Results

### Description of the Study Sample

Study participants (*n = *2,948) were on average 19.4 (SD = 1.9) years old when completing the fall surveys and were most commonly in their first year of undergraduate studies (40%) (Table 1). The 2021/22 academic year was most represented (45%), followed by 2023/24 (29%) and 2022/23 (26%). Most students were domestic (95%), and identified as female (76%), White (61%) or East/Southeast Asian ethnicity (16%), and heterosexual (70%). Nearly one-third (31%) reported a lifetime history of a diagnosed mental illness. Most students were studying Health, Life & Physical Sciences (36%) or Arts, Humanities, & Social Sciences (31%).

**Table 1.** Description of the Combined (2021–2022 to 2023–2024) Study Sample (*n = *2,948).

	*n*	(%)
Age at the start of the academic year, *Mean (SD)*	*19.4 (1.9)*	
≤18	1099	(38.9)
19	553	(19.6)
20-21	944	(33.4)
22+	230	(8.1)
Year of study		
First	1189	(40.3)
Second	670	(22.7)
Third	554	(18.8)
Fourth	535	(18.2)
Gender		
Female	2215	(76.3)
Male	595	(20.5)
Non-binary^a^	95	(3.3)
Sexual orientation		
Heterosexual	1638	(70.4)
2SLGBTQIA+^b^	690	(29.6)
International student, *Yes*	151	(5.2)
Ethnicity		
White	1757	(60.8)
East/Southeast Asian	463	(16.0)
South Asian	163	(5.6)
Middle Eastern	76	(2.6)
Black	52	(1.8)
Other ethnicity (including multiple)	381	(13.2)
History of diagnosed mental illness, Yes	852	(30.5)
Academic program		
Health, life and physical sciences	1065	(36.3)
Arts, humanities, and social sciences	915	(31.2)
Professional schools^c^	440	(15.0)
Engineering and applied science	421	(14.3)
Computing	96	(3.3)
Parental education*, highest completed*		
Advanced degree^d^	1173	(41.0)
Bachelor's or trades/apprentice	1311	(45.8)
High school or less	377	(13.2)
Any unacceptable experiences, *Yes*	852	(28.9)
Number of different unacceptable Experiences		
None	2096	(71.1)
1	511	(17.3)
2	212	(7.2)
3	74	(2.5)
4	29	(1.0)
5	26	(0.9)

^a^ Non-binary: includes non-binary and prefer not to say.

^b^ 2SLGBTQIA+: Homosexual/Lesbian/Gay/Bisexual, No label, Prefer not to say, Other.

^c^ Professional Schools: Law, Business/Commerce, Medicine, Nursing.

^d^ Advanced Degree: Doctorate/Professional Degree and Master's.

### Description of Unacceptable Experiences (UEs)

Almost one-third of students (28.9%; *n = *852/2,948) reported having experienced at least one of the UEs over the academic year (Table 1). Further, 17% and 7% reported two or three different UEs, respectively. Among students who participated in multiple academic years (*n = *596), those who reported a UE in the first year were significantly more likely to report a UE in the subsequent year (49.7% vs. 15.4%, *P* < .001). Discrimination (14.6%) and sexual violence or harassment (14.4%) were the most reported experiences, followed by bullying/harassment (10.6), hate crimes (5.0%) and physical assault (3.2%) (Table 2). Correlations between the experiences ranged from 0.17 to 0.41. The most strongly correlated were discrimination and hate crimes (Spearman's rho (*r*s) = 0.41), bullying and hate crimes (*r*s = 0.34) and bullying and discrimination (*r*s = 0.32).

**Table 2.** Frequency of Unacceptable Experiences (Yes or Unsure), by Demographic Characteristics.

	n Total^a^	Discrimination		Sexual violence or harassment		Bullying/harassment		Hate crime		Physical assault	
		%Yes	(95% CI)	%Yes	(95% CI)	%Yes	(95% CI)	%Yes	(95% CI)	%Yes	(95% CI)
Overall	2948	14.6	(13.1–16.0)	14.4	(13.0–15.8)	10.6	(9.4–11.8)	5.0	(4.1–5.8)	3.2	(2.5–3.9)
Age at the start of the academic year											
≤18	1099	15.5	(13.3–17.7)	17.0	(14.7–19.2)	12.0	(10.0–13.9)	5.2	(3.9–6.6)	4.0	(2.9–5.2)
19	553	13.4	(10.6–16.3)	12.7	(9.9–15.4)	9.1	(6.7–11.5)	4.7	(2.9–6.5)	2.5	(1.2–3.8)
20–21	944	14.3	(11.8–16.8)	14.4	(12.0–16.8)	9.9	(7.9–11.9)	5.1	(3.7–6.5)	3.2	(2.0–4.3)
22+	230	12.7	(8.2–17.2)	7.8	(4.2–11.5)	11.7	(7.3–16.2)	4.8	(1.8–7.8)	2.2	(0.3–4.1)
Gender											
Female	2251	14.6	(12.9–16.3)	16.6	(14.9–18.3)	10.8	(9.4–12.2)	4.9	(3.9–5.9)	3.3	(2.5–4.1)
Male	595	11.7	(8.7–14.6)	4.7	(2.9–6.5)	8.8	(6.2–11.4)	4.7	(2.9–6.6)	3.4	(1.9–4.8)
Non–binary	95	32.6	(21.2–44.1)	22.1	(12.7–31.6)	20.2	(11.6–28.8)	10.5	(4.5–16.6)	2.1	(0–5.1)
Sexual orientation											
Heterosexual	1638	11.4	(9.7–13.2)	11.2	(9.6–12.9)	9.3	(7.8–10.8)	4.3	(3.3–5.4)	2.9	(2.0–3.7)
2SLGBTQIA+	690	21.2	(17.6–24.9)	22.2	(18.7–25.7)	14.6	(11.7–17.4)	7.0	(4.9–9.1)	3.6	(2.0–5.2)
International/domestic status											
Domestic	2736	14.4	(12.9–15.9)	14.6	(13.2–16.1)	10.8	(9.6–12.1)	5.0	(4.1–5.8)	3.2	(2.5–3.9)
International	151	17.5	(11.0–23.9)	9.9	(4.6–15.3)	8.7	(3.2–14.1)	6.7	(2.3–11.0)	4.0	(0.8–7.2)
Ethnicity											
White	1757	8.6	(7.1–10.1)	16.6	(14.6–18.5)	10.5	(9.0–12.0)	3.2	(2.3–4.1)	3.6	(2.7–4.6)
Black	52	32.7	(17.6–47.8)	15.4	(5.0–25.8)	11.5	(2.6–20.5)	13.5	(2.6–24.4)	0	(0)
Middle Eastern	76	23.7	(13.6–33.7)	9.2	(1.7–16.8)	11.8	(3.7–20.0)	10.5	(3.7–17.4)	2.6	(0–6.4)
East/ Southeast Asian	463	24.2	(19.6–28.7)	7.6	(5.0–10.2)	9.5	(6.7–12.4)	8.0	(5.3–10.8)	2.4	(1.0–3.8)
South Asian	163	22.4	(15.0–29.7)	8.0	(3.5–12.5)	9.9	(5.2–14.6)	6.2	(2.4–10.0)	3.1	(0.4–5.8)
Other ethnicity	381	23.1	(18.1–28.1)	16.1	(12.1–20.0)	13.2	(9.2–17.1)	7.1	(4.3–10.0)	2.9	(1.2–4.6)
Lifetime history of mental illness											
No history	1941	12.7	(11.0–14.3)	10.9	(9.4–12.4)	7.9	(6.6–9.2)	4.1	(3.2–5.1)	2.4	(1.7–3.1)
Yes	852	19.3	(16.1–22.4)	23.2	(20.0–26.3)	17.2	(14.4–19.9)	7.1	(5.2–8.9)	5.2	(3.5–6.8)
Academic program											
Arts, humanities, social sciences	915	15.0	(12.3–17.6)	19.2	(16.3–22.0)	12.3	(9.9–14.6)	6.2	(4.5–8.0)	3.8	(2.5–5.2)
Health, life and physical sciences	1065	14.9	(12.3–17.4)	14.0	(11.7–16.3)	11.5	(9.4–13.5)	4.6	(3.3–5.9)	3.5	(2.3–4.6)
Engineering and applied science	421	15.5	(11.6–19.3)	10.9	(7.7–14.2)	8.4	(5.6–11.1)	4.0	(2.0–6.1)	2.6	(1.1–4.1)
Computing	96	12.8	(5.5–20.0)	5.2	(0.8–9.7)	2.1	(0–5.0)	3.2	(0–6.7)	3.2	(0–6.7)
Professional schools	440	12.7	(9.2–16.3)	10.5	(7.4–13.5)	9.3	(6.4–12.2)	4.6	(2.5–6.6)	1.8	(0.6–3.1)
Parental education*, highest completed*											
Advanced degree	1173	15.5	(13.2–17.9)	13.0	(10.8–15.1)	10.5	(8.6–12.5)	4.6	(3.3–5.9)	3.0	(1.9–4.1)
Bachelor's or Trades/apprentice	1311	12.4	(10.3–14.4)	14.4	(12.3–16.5)	9.9	(8.2–11.6)	4.4	(3.2–5.7)	3.2	(2.2–4.2)
High school or less	377	19.0	(14.5–23.5)	19.6	(15.4–23.9)	13.9	(9.9–17.8)	8.0	(5.0–11.0)	4.0	(2.0–6.0)

^a^ ≤ 5.1% missing by demographic characteristic and unacceptable experience; sexual orientation was added to the baseline survey in Fall 2020, and was not captured for *n* = 587 students who entered the study in 2018 and 2019.

**Figure 1.**
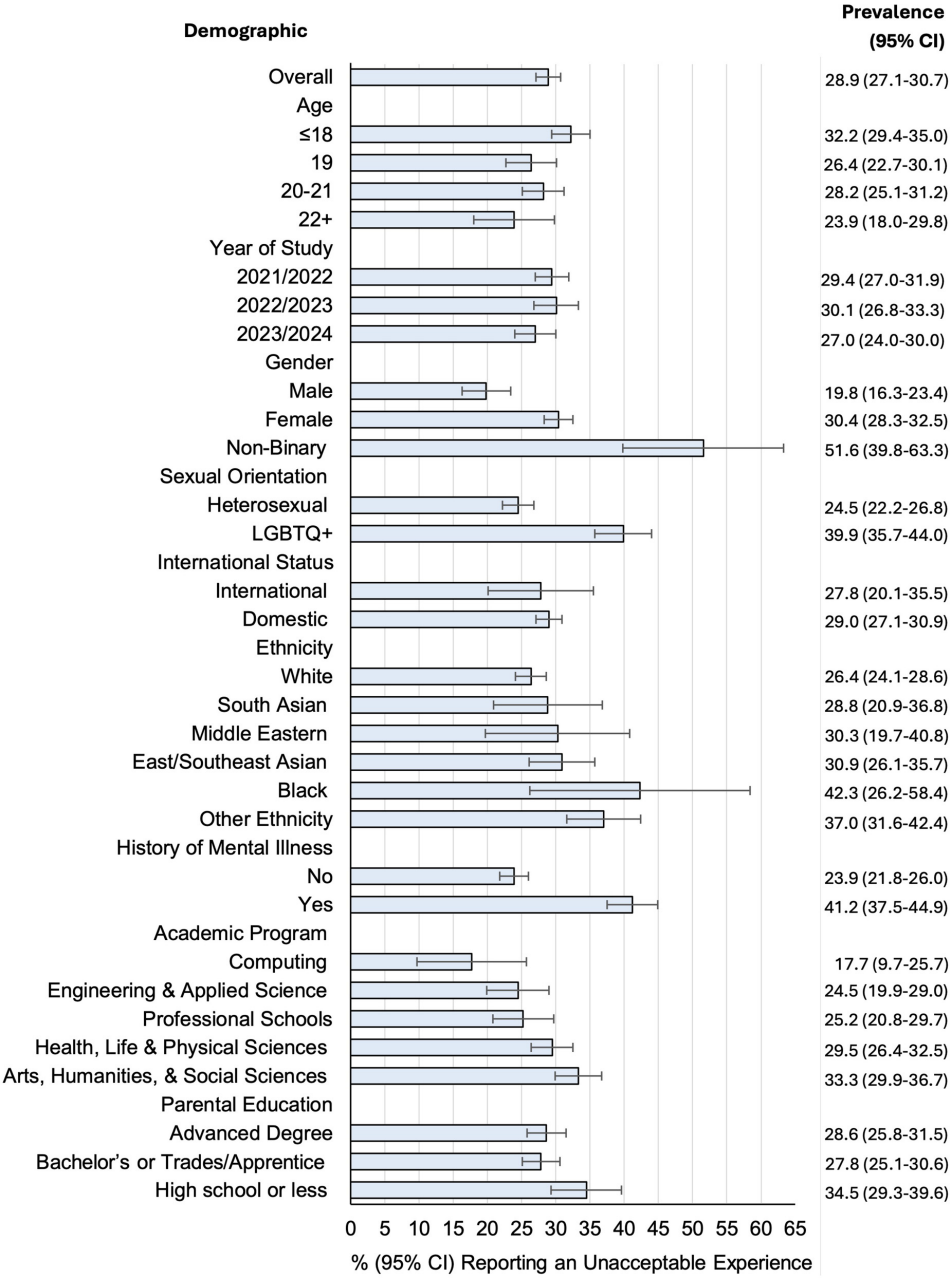
Proportion of university students reporting being the victim of an unacceptable experience over the academic year, overall and by demographic subgroup.

Figure 1 displays the proportion of students reporting a UE over the academic year, in the full sample and by demographic subgroup. UEs were most commonly reported by students from minoritised subgroups identifying as non-binary gender (51.6%), 2SLGBTQIA + (39.9%), Black (42.3%) and those with a lifetime mental illness (41.2%) (Figure 1). A greater proportion of females reported a UE compared to males (30.4% vs. 19.8%, *P* < .001). Further, UEs were more commonly reported by students with lower parental education (i.e., high school or less) (34.5% vs. 28.2%, *P* = .02) and less commonly reported by students in Computing Sciences compared to other programs (17.7% vs. 29.3%, *P* = .02). Reporting of UEs was comparable across groups defined by international student status and academic year (Figure 1).

Sexual violence or harassment was more commonly reported by students identifying as female (16.6%) or non-binary gender (22.1%) compared to male (4.7%, *P* < .001), 2SLGBTQIA + compared to those identifying as heterosexual (22.2% vs. 11.2%, *P* < .001) and students reporting a lifetime mental illness (23.2% vs. 10.9%, *P* < .001) (Table 2). Sexual violence or harassment was also more commonly reported by students who were younger than 19 at the start of the year (17.0% vs. 13.0%, *P* = .004) and had a parental education level of high school or less (19.6% vs. 13.7%, *P* = .005). It was less common in those identifying as Asian or Middle Eastern compared to other ethnicities (7.9% vs. 16.5%, *P* < .001) (Table 2)**.**

Discrimination was more commonly reported among students from minoritised subgroups based on gender (non-binary vs. male or female 32.6% vs. ≤14.6%, *P* < .001), sexual orientation (21.2% 2SLGBTQIA + vs. 11.4% heterosexual students, *P* < .001) and ethnicity (8.6% white vs. 23.9% other ethnicities, *P* < .001) (Table 2). The prevalence of physical assault was relatively low across student subgroups but was most frequently reported by those with a lifetime mental illness (5.2%). Additionally, hate crimes and bullying/harassment were reported more among non-binary students than students identifying as male or female (10.5% vs. ≤4.9%, *P* = .03 and 20.2% vs. ≤10.8%, *P* ≤ .01, respectively), and hate crimes were more frequently reported among students from ethnic minority subgroups compared to students identifying as white (7.9% vs. 3.2%, *P* < .001).

### UEs and Mental Health Problems

At the end of the academic year, 43% of students surveyed met screening thresholds for anxiety, 42% for depression and 26.3% for low well-being, and 17% endorsed suicidal thoughts or behaviours over the academic year. With few exceptions, students reporting a UE over the academic year were significantly more likely to screen positive for a mental health concern at the end of the year (Table 3). Apart from hate crimes, which had a non-significant effect in the same direction, UEs had a statistically significant association with increased risk of reporting suicidal thoughts and/or behaviours. Each UE was associated with an increased risk of screening positive for anxiety (21–24% more likely) and depression (26–46% more likely). After adjusting for baseline symptoms, the associations between UEs and anxiety were reduced (10–19% increased risk), but the associations with depression largely remained (14–40% increased risk). Associations between UE exposure and mental health concerns were strongest for physical assault followed by sexual violence or harassment and discrimination. Physical assault, bullying/harassment, hate crimes and discrimination were associated with an increased risk of reporting low well-being at the end of the year after accounting for baseline well-being levels (28–39%; Table 3). Results from the multiple imputation analyses were consistent with those from the complete case analysis, except for the associations between hate crimes and suicidal thoughts and behaviours, and between sexual violence or harassment and low well-being, where the fully adjusted relative risks increased by 11% and 7%, respectively. In addition, the relative risks for physical assault increased by 5–8% in the fully adjusted models.

**Table 3.** Results of Multivariable Log-Binomial Regression Analyses Examining Associations Between Unacceptable Experiences Over the Academic Year and Mental Health and Well-Being at the End of the Academic Year.

	Anxiety (GAD–7 ≥ 10)						Depression (PHQ–9 ≥ 10)					
	n Total	%Yes	Model 1^a^		Model 2^b^		n Total	%Yes	Model 1^a^		Model 2^b^	
			RR	(95% CI)	RR	(95% CI)			RR	(95% CI)	RR	(95% CI)
Sexual violence or harassmen**t**												
No	1995	39.8	1.00	ref	1.00	ref	1981	38.9	1.00	ref	1.00	ref
Yes/not sure	351	62.4	1.22	(1.10–1.36)	1.10	(1.00–1.21)	349	63.3	1.33	(1.19–1.49)	1.22	(1.11–1.34)
Discrimination												
No	2002	41.5	1.00	ref	1.00	ref	1990	39.6	1.00	ref	1.00	ref
Yes/not sure	345	53.3	1.21	(1.08–1.37)	1.11	(0.99–1.24)	341	60.4	1.37	(1.23–1.52)	1.27	(1.15–1.41)
Bullying/harassment												
No	2088	41.0	1.00	ref	1.00	ref	2074	40.1	1.00	ref	1.00	ref
Yes/not sure	256	60.6	1.24	(1.11–1.38)	1.12	(1.01–1.24)	254	63.0	1.29	(1.14–1.45)	1.14	(1.03–1.27)
Hate crimes												
No	2232	42.4	1.00	ref	1.00	ref	2218	41.7	1.00	ref	1.00	ref
Yes/not sure	112	57.1	1.23	(1.06–1.42)	1.16	(1.00–1.34)	110	60.0	1.26	(1.10–1.45)	1.17	(1.01–1.36)
Physical assault												
No	2270	42.4	1.00	ref	1.00	ref	2255	41.4	1.00	ref	1.00	ref
Yes/not sure	75	65.3	1.24	(1.04–1.48)	1.19	(1.02–1.38)	74	77.0	1.46	(1.25–1.71)	1.40	(1.20–1.62)
	Low well–being (SWEMWBS ≤19)						Suicidal thoughts and behaviours (past 6 months)					
			Model 1^a^		Model 2^b^				Model 1^a^		Model 2^b^	
	n Total	%Yes	RR	(95% CI)	RR	(95% CI)	n Total	%Yes	RR	(95% CI)	RR	(95% CI)
Sexual violence or harassment											Current (past 6 month) suicidal thoughts and behaviours not available on all versions of the Fall baseline survey	
No	2012	25.6	1.00	ref	1.00	ref	2289	14.9	1.00	ref		
Yes/not sure	353	35.4	1.15	(0.97–1.36)	1.04	(0.89–1.20)	401	33.7	1.72	(1.45–2.05)		
Discrimination												
No	2017	25.2	1.00	ref	1.00	ref	2298	15.6	1.00	ref		
Yes/not sure	349	37.8	1.36	(1.17–1.60)	1.28	(1.11–1.47)	391	30.2	1.64	(1.37–1.96)		
Bullying/harassment												
No	2104	24.8	1.00	ref	1.00	ref	2399	15.8	1.00	ref		
Yes/not sure	259	45.6	1.59	(1.36–1.85)	1.37	(1.19–1.57)	288	34.0	1.67	(1.39–2.01)		
Hate crimes												
No	2250	26.4	1.00	ref	1.00	ref	2553	17.3	1.00	ref		
Yes/not sure	113	40.7	1.34	(1.05–1.72)	1.30	(1.05–1.61)	134	26.1	1.13	(0.82–1.56)		
Physical assault												
No	2288	26.4	1.00	ref	1.00	ref	2600	17.0	1.00	ref		
Yes/not sure	76	48.7	1.46	(1.12–1.92)	1.39	(1.10–1.74)	88	36.4	1.56	(1.10–2.22)		

^a^ Model 1 Adjusted for age, gender, lifetime history of mental illness, ethnicity, and parental education level.

^b^ Model 2 Adjusted for age, gender, lifetime history of mental illness, ethnicity, parental education level, and mental health status at baseline.

Students who reported a UE were more likely to access campus mental health and wellness services over the year (36% vs. 21%, *P* < .001), ranging from 37% of students who reported experiencing discrimination to 44% for physical assault, and 45% for those reporting a hate crime

### UEs and Academic Performance

The average cumulative GPA over the year was 3.41 (SD = 0.83) in the full sample and 3.37 (SD = 0.79) in first-year students. In the full sample, sexual violence or harassment was associated with a 0.09 (95% CI: −0.17, −0.01) decrease in average cumulative GPA (Table 4). The other UEs were not significantly associated with GPA after adjustments, although having experienced bullying/harassment had evidence of a borderline association with a 0.08-point lower (95% CI: −0.17, 0.01) average GPA (Table 4). However, among first-year undergraduate students specifically, there was evidence that hate crimes (β= −0.25; −0.45, −0.04) and bullying/harassment (β= −0.14; −0.28, −0.00) had significant negative effects on cumulative GPA, and a reported experience of sexual violence or harassment (β= −0.12; −0.24, 0.01) had a greater negative effect than in the full sample of undergraduate students (Table 4). The multiple imputation results were consistent with those presented except for the negative effect of bullying/harassment in the full sample, which increased in magnitude and became statistically significant (β = −0.16; 95% CI: −0.27, −0.05; *P* *=* .01).

**Table 4.** Results of Multivariable Linear Regression Examining Associations Between Unacceptable Experiences and Academic Performance (Cumulative GPA).

	All students						First year students					
	*n*	GPA					*n*	GPA				
		*M*	(SD)	β	(95% CI)	*P*		*M*	(SD)	β	(95% CI)	*P*
Sexual violence or harassment												
No	2194	3.44	(0.84)	ref	/		882	3.41	(0.77)	ref	/	
Yes	396	3.33	(0.82)	−0.09	(−0.17, −0.01)	.04	176	3.20	(0.84)	−0.12	(−0.24, 0.01)	.06
Discrimination												
No	2210	3.42	(0.82)	ref	/		897	3.39	(0.75)	ref	/	
Yes	378	3.36	(0.94)	−0.04	(−0.12, 0.05)	.35	162	3.26	(0.96)	−0.07	(−0.20, 0.06)	.27
Bullying/harassment												
No	2304	3.44	(0.82)	ref	/		926	3.40	(0.76)	ref	/	
Yes	282	3.27	(0.90)	−0.08	(−0.17, 0.01)	.09	132	3.18	(0.95)	−0.14	(−0.28, −0.00)	.04
Hate crime												
No	2459	3.43	(0.83)	ref	/		1000	3.39	(0.77)	ref	/	
Yes	127	3.28	(0.96)	−0.06	(−0.20, 0.07)	.35	56	3.08	(1.02)	−0.25	(−0.45, −0.04)	.02
Physical assault												
No	2500	3.42	(0.84)	ref	/		1018	3.38	(0.79)	ref	/	
Yes	87	3.38	(0.73)	−0.03	(−0.20, 0.14)	.73	39	3.23	(0.82)	−0.16	(−0.40, 0.08)	.19
Any experience												
No	1822	3.44	(0.82)	ref	/		718	3.41	(0.76)	ref	/	
Yes	770	3.37	(0.86)	−0.05	(−0.11, 0.02)	.15	342	3.30	(0.84)	−0.04	(−0.14, 0.06)	.42

Note: Models adjusted for age, gender, lifetime history of mental illness, parental education, ethnicity, and program of study

## Discussion

Nearly 30% of undergraduate students attending a major Canadian university reported at least one UE in each of three consecutive academic years from 2021/2022 to 2023/2024. Discrimination and sexual violence or harassment were the most frequently reported UEs, each affecting 14–15% of students surveyed, followed by bullying or harassment (11%). Students who identified as female, non-binary gender, or 2SLGBTQIA+, those belonging to a minority ethnic group, and those with a lifetime mental illness more frequently reported experiencing UEs. A significant association was found between reported UEs and increased risk of screening positive for anxiety, depression, low well-being and suicidal thoughts and behaviours. Further, reported sexual violence or harassment and, among first-year undergraduates, bullying and hate crimes were associated with lower cumulative GPA.

### Sexual Orientation and Gender

Approximately 50% of students identifying as 2LSGBTQIA + and/or non-binary gender reported experiencing a UE over the academic year. This finding aligns with the minority stress theory, which posits that persons belonging to minority groups are more susceptible to experiencing negative life events and associated stress,^32–34^ often associated with challenges related to individuation, self-acceptance, stigma and social rejection.^35,36^ In addition to being at greater risk of UEs, students identifying as transgender, genderqueer, or genderfluid are also more vulnerable to the psychological impact of these exposures.^37,38^ For example, non-cisgender youth are three times more likely than cisgender youth to report a suicide attempt, and are more susceptible to substance misuse, and emotional distress from bullying.^39^

Sexual violence has consistently been demonstrated to be a form of gender-based abuse that disproportionately affects women.^40^ This is consistent with our findings that female students were over three times more likely than males to report sexual violence or harassment, but were equally as likely to report other forms of UEs. Additionally, as most sexual assault campaigns are centred around de-stigmatising reporting among female victims, there could be greater willingness to disclose these types of experiences.^41^ More subtle forms of sexual abuse, such as sexual coercion, which are more commonly reported by heterosexual females in Canadian universities, were likely under-reported in this study, as they were not specifically asked about.^42^

### Ethnic Minorities

Although we did not directly examine effect modification by gender, sexuality, or race/ethnicity due to sample size limitations, our findings suggest that minority subgroups are disproportionately affected. For example, students identifying as Black and other minoritised ethnic identities on campus were 10% more likely to report a UE compared to students identifying as White or Asian. Higher education students from ethnic minorities have previously been shown to have an increased likelihood of reporting microaggression, racism and discrimination.^43^ A 2024 scoping review of systemic racism in Canadian higher education identified themes reported by ethnic minority students, particularly those identifying as Black and Indigenous, including feelings of marginalisation and exclusion from social life on campus, which in turn were associated with decreased participation in classes and increased vulnerability to UEs. While there is some evidence that overt forms of racism and discrimination have declined, more subtle forms such as microaggression remain a concern.^43^ These subtle forms of discrimination have been linked to chronic stress and negative health outcomes among ethnic minority groups but the mechanisms through which this occurs are poorly understood.^7^

### Lifetime History of Mental Illness

Students with a lifetime mental illness were almost twice as likely to report a UE than students without this history. A personal history of mental illness has long been established as a risk factor predicting both exposure to more adverse life events, including UEs, and greater responsivity to such adverse exposures.^44^ These individuals also have a greater likelihood of experiencing compounding effects from stigmatisation and social exclusion.^45^ This aligns with the model of gene-environment interactive effects, in which those predisposed genetically to a mental illness are also more likely to experience adverse events and associated stress and distress reflecting more sensitive biological (i.e., genetic, epigenetic) and cognitive-emotional pathways.^46,47^

### Mental Health Impact

Consistent with findings of previous studies, students who reported a UE while studying at university were at increased risk of experiencing clinically significant symptoms of anxiety, depression, and suicidal thoughts and behaviours.^2^ Prior research has reported an association between sexual assault and higher anxiety and depressive symptoms three and six months later in university students.^48^ Further, exposure to bullying in adolescence is associated with an increased risk of depression and anxiety disorders and suicidality in young adulthood.^14^ US college students who reported experiencing discrimination based on their race/ethnicity, sexual orientation, or gender in the past year were 35–39% more likely to report symptoms of mental health problems, including anxiety, depression and suicidality, and had 88–99% increased odds of being diagnosed with a mental disorder.^49^ Therefore, prior research aligns with the findings of the current study and underscores the enduring impact UEs can have on the mental health of university students.

### Academic Performance

Sexual violence and bullying/harassment were modestly associated with lower academic performance in the full undergraduate sample. However, in first-year students transitioning to university, there was evidence of a significant negative effect of sexual violence, bullying, and hate crimes on cumulative GPA over the academic year. These results align with literature indicating campus violence and discrimination have a negative impact on academic success, particularly for students from gender, sexual and ethnic minority groups.^50,51^ Moreover, anxiety and depressive symptoms are associated with lower academic performance,^31^ suggesting UEs may negatively impact academic performance through their impact on student mental health. Although the observed effects on GPA were generally modest, they were more pronounced for first-year students, as evidenced by a 0.25 average decrease in GPA associated with hate crimes. Such differences during the transition to university can significantly impact students’ future opportunities, including admission to competitive postgraduate programs, entry into the workforce, and eligibility for academic scholarships. Increased help-seeking in individuals reporting UEs found in this study, may have provided a protective effect on mental health and academic performance, as evidenced in some studies showing a positive association between counselling and academic success.^52^

### Strengths and Limitations

The U-Flourish survey captured large cohorts of diverse undergraduate students attending a major Canadian University across three consecutive years. Using this data, we were able to examine the prevalence of different UEs across student subgroups. Well-validated measures assessed mental health outcomes, and objective GPA data was obtained from university administrative data. Nonetheless, certain limitations should be considered including reliance on self-report for capturing UEs potentially introducing recall error and information bias. Female students were overrepresented in the sample (76%), which may have inflated overall prevalence estimates, given that several of the UEs were more commonly reported by females than males. The relatively small number of male participants may limit the representativeness of their experiences. Given this was a single-site study at a Canadian university it may limit generalisability to campuses with different demographics, such as francophone or Indigenous-serving institutions, or universities in more urban settings. Although we adjusted for baseline mental health, temporal ambiguity remains because the exact timing of UEs relative to changes in mental health could not be determined. As with any voluntary survey, non-response and attrition may have resulted in participation bias. Finally, we were unable to assess the prior impact of UEs, and information on the nature, severity or frequency of the UEs was not available.

### Implications and Future Directions

The transition to university is a critical period for psychosocial development; a time when individuals refine their self-concept, beliefs and principles, and gain advanced cognitive skills to navigate complex life experiences.^53^ However, UEs, such as discrimination and sexual violence, can disrupt this developmental process, have a substantive negative impact on mental health, and derail academic performance. Moreover, longitudinal studies suggest the effects of such adverse experiences on mental health can persist for years following initial exposure.^54,55^ Future research should aim to elucidate the mechanisms underlying these exposures and their impact through an intersectional lens, considering factors such as race, gender, previous history of mental illness and early adversity (prior UEs and childhood abuse). This understanding would be essential for effective universal and targeted prevention. Students who experienced UEs reported higher rates of service use, highlighting the importance of evaluating the accessibility, effectiveness, and cultural responsiveness of campus mental health and reporting systems. Further, preliminary findings around help-seeking underscores the need for further investigation into the effectiveness of support received and barriers and inequities for students at risk for and having experienced UEs. Through utilising reliable data to improve understanding, more targeted prevention and early intervention initiatives for postsecondary students can be developed. Addressing these issues will require sustained partnership and involvement of students, especially those from high-risk subgroups, to achieve and foster a campus culture that promotes and provides a safe and inclusive learning community.

## Conclusion

Nearly 30% of undergraduates attending a major Canadian university reported at least one unacceptable experience over the academic year across a three-year period, with certain identifiable subgroups disproportionately affected. Students identifying as female and non-binary gender were more likely to report sexual violence or harassment, while those with a lifetime mental illness, identifying as 2SLGBTQIA+, and students from ethnic minorities also reported higher rates of UEs. These experiences had a detrimental impact on student mental health and were associated with moderate declines in academic performance, especially in first year students. The development of universal campus-wide prevention to create a safe and welcoming learning community and culturally informed targeted intervention to address and mitigate the adverse effects of UEs is indicated.

## Supplemental Material

sj-docx-1-cpa-10.1177_07067437251412566 - Supplemental material for Unacceptable Experiences Reported by Undergraduate Students and Their Associations With Mental Health, Well-Being and Academic Performance: U-Flourish Student Well-Being Research: Expériences inacceptables signalées par les étudiants de premier cycle et leurs liens avec la santé mentale, le bien-être et le rendement académique : Programme de recherche U-Flourish sur le bien-être des étudiants Supplemental material, sj-docx-1-cpa-10.1177_07067437251412566 for Unacceptable Experiences Reported by Undergraduate Students and Their Associations With Mental Health, Well-Being and Academic Performance: U-Flourish Student Well-Being Research: Expériences inacceptables signalées par les étudiants de premier cycle et leurs liens avec la santé mentale, le bien-être et le rendement académique : Programme de recherche U-Flourish sur le bien-être des étudiants by S. Hayden John, Anne Duffy, Alice Y.S. Li, Kristen Kyone, Emily Dephoure, Daniel Rivera, Adeleine Lyon and Nathan King in The Canadian Journal of Psychiatry

sj-docx-2-cpa-10.1177_07067437251412566 - Supplemental material for Unacceptable Experiences Reported by Undergraduate Students and Their Associations With Mental Health, Well-Being and Academic Performance: U-Flourish Student Well-Being Research: Expériences inacceptables signalées par les étudiants de premier cycle et leurs liens avec la santé mentale, le bien-être et le rendement académique : Programme de recherche U-Flourish sur le bien-être des étudiants Supplemental material, sj-docx-2-cpa-10.1177_07067437251412566 for Unacceptable Experiences Reported by Undergraduate Students and Their Associations With Mental Health, Well-Being and Academic Performance: U-Flourish Student Well-Being Research: Expériences inacceptables signalées par les étudiants de premier cycle et leurs liens avec la santé mentale, le bien-être et le rendement académique : Programme de recherche U-Flourish sur le bien-être des étudiants by S. Hayden John, Anne Duffy, Alice Y.S. Li, Kristen Kyone, Emily Dephoure, Daniel Rivera, Adeleine Lyon and Nathan King in The Canadian Journal of Psychiatry

sj-docx-3-cpa-10.1177_07067437251412566 - Supplemental material for Unacceptable Experiences Reported by Undergraduate Students and Their Associations With Mental Health, Well-Being and Academic Performance: U-Flourish Student Well-Being Research: Expériences inacceptables signalées par les étudiants de premier cycle et leurs liens avec la santé mentale, le bien-être et le rendement académique : Programme de recherche U-Flourish sur le bien-être des étudiants Supplemental material, sj-docx-3-cpa-10.1177_07067437251412566 for Unacceptable Experiences Reported by Undergraduate Students and Their Associations With Mental Health, Well-Being and Academic Performance: U-Flourish Student Well-Being Research: Expériences inacceptables signalées par les étudiants de premier cycle et leurs liens avec la santé mentale, le bien-être et le rendement académique : Programme de recherche U-Flourish sur le bien-être des étudiants by S. Hayden John, Anne Duffy, Alice Y.S. Li, Kristen Kyone, Emily Dephoure, Daniel Rivera, Adeleine Lyon and Nathan King in The Canadian Journal of Psychiatry
