# Supplementary material for: Unacceptable Experiences Reported by Undergraduate Students and Their Associations With Mental Health, Well-Being and Academic Performance: U-Flourish Student Well-Being Research: Expériences inacceptables signalées par les étudiants de premier cycle et leurs liens avec la santé mentale, le bien-être et le rendement académique : Programme de recherche U-Flourish sur le bien-être des étudiants
Source: Can J Psychiatry. 2026 Feb 10:07067437251412566. Online ahead of print. doi: 10.1177/07067437251412566 (PMC12890602; doi:10.1177/07067437251412566)
Supplement: sj-docx-1-cpa-10.1177_07067437251412566 - Supplemental material for Unacceptable Experiences Reported by Undergraduate Students and Their Associations With Mental Health, Well-Being and Academic Performance: U-Flourish Student Well-Being Research: Expériences inacceptables signalées par les étudiant [file sj-docx-1-cpa-10.1177_07067437251412566.docx]

| **Supplementary Table 1A**. Results of multivariable log-binomial regression analyses examining associations between unacceptable experiences (yes, no, or not sure) reported over the academic year and clinically significant symptoms of anxiety and depression reported at the end of the academic year | | | | | | | | | | | | | |
| --- | --- | --- | --- | --- | --- | --- | --- | --- | --- | --- | --- | --- | --- |
|  | | **Anxiety (GAD-7 ≥10)** | | | | | | **Depression (PHQ-9 ≥10)** | | | | | |
|  |  | **nTot** | **%Yes** | **Model 1*** | | **Model 2**** | | **nTot** | **%Yes** | **Model 1*** | | **Model 2**** | |
|  |  |  |  | **RR** | **(95% CI)** | **RR** | **(95% CI)** |  |  | **RR** | **(95% CI)** | **RR** | **(95% CI)** |
| **Sexual Violence or Harassment** | | | |  |  |  |  |  |  |  |  |  |  |
|  | No | 1995 | 39.8 | 1.00 | ref | 1.00 | ref | 1981 | 38.9 | 1.00 | ref | 1.00 | ref |
|  | Yes | 239 | 62.8 | 1.21 | (1.07-1.36) | 1.07 | (0.97-1.19) | 238 | 64.3 | 1.35 | (1.18-1.53) | 1.22 | (1.10-1.35) |
|  | Not Sure | 112 | 61.6 | 1.25 | (1.05-1.48) | 1.18 | (1.01-1.37) | 111 | 61.3 | 1.30 | (1.11-1.52) | 1.23 | (1.06-1.43) |
| **Discrimination** | |  |  |  |  |  |  |  |  |  |  |  |  |
|  | No | 2002 | 41.5 | 1.00 | ref | 1.00 | ref | 1990 | 39.6 | 1.00 | ref | 1.00 | ref |
|  | Yes | 192 | 60.4 | 1.32 | (1.16-1.52) | 1.19 | (1.04-1.36) | 190 | 60.5 | 1.39 | (1.22-1.58) | 1.22 | (1.08-1.37) |
|  | Not Sure | 153 | 44.4 | 1.07 | (0.90-1.27) | 1.00 | (0.84-1.19) | 151 | 60.3 | 1.35 | (1.18-1.54) | 1.35 | (1.19-1.53) |
| **Bullying/Harassment** | | |  |  |  |  |  |  |  |  |  |  |  |
|  | No | 2088 | 41.0 | 1.00 | ref | 1.00 | ref | 2074 | 40.1 | 1.00 | ref | 1.00 | ref |
|  | Yes | 172 | 66.3 | 1.29 | (1.15-1.45) | 1.18 | (1.05-1.32) | 172 | 65.7 | 1.33 | (1.16-1.51) | 1.14 | (1.01-1.29) |
|  | Not Sure | 84 | 48.8 | 1.11 | (0.90-1.36) | 0.98 | (0.81-1.19) | 82 | 57.3 | 1.18 | (0.95-1.48) | 1.15 | (0.96-1.38) |
| **Hate crimes** | |  |  |  |  |  |  |  |  |  |  |  |  |
|  | No | 2232 | 42.4 | 1.00 | ref | 1.00 | ref | 2218 | 41.7 | 1.00 | ref | 1.00 | ref |
|  | Yes | 64 | 57.8 | 1.18 | (0.97-1.42) | 1.08 | (0.91-1.30) | 63 | 61.9 | 1.21 | (1.02-1.44) | 1.12 | (0.94-1.34) |
|  | Not Sure | 48 | 56.3 | 1.30 | (1.04-1.62) | 1.28 | (1.02-1.61) | 47 | 57.5 | 1.33 | (1.07-1.64) | 1.25 | (0.97-1.61) |
| **Physical Assault** | |  |  |  |  |  |  |  |  |  |  |  |  |
|  | No | 2270 | 42.4 | 1.00 | ref | 1.00 | ref | 2255 | 41.4 | 1.00 | ref | 1.00 | ref |
|  | Yes | 44 | 77.3 | 1.37 | (1.12-1.67) | 1.22 | (1.02-1.44) | 44 | 81.8 | 1.49 | (1.25-1.79) | 1.37 | (1.16-1.62) |
|  | Not Sure | 31 | 48.4 | 1.04 | (0.73-1.46) | 1.13 | (0.84-1.51) | 30 | 70.0 | 1.41 | (1.09-1.83) | 1.44 | (1.10-1.89) |
| **Model 1 Adjusted for age, gender, lifetime history of mental illness, ethnicity, and parental education level*  ***Model 2 Adjusted for age, gender, lifetime history of mental illness, ethnicity, parental education level, and mental health status at baseline* | | | | | | | | | | | | | |
