# Supplementary material for: Unacceptable Experiences Reported by Undergraduate Students and Their Associations With Mental Health, Well-Being and Academic Performance: U-Flourish Student Well-Being Research: Expériences inacceptables signalées par les étudiants de premier cycle et leurs liens avec la santé mentale, le bien-être et le rendement académique : Programme de recherche U-Flourish sur le bien-être des étudiants
Source: Can J Psychiatry. 2026 Feb 10:07067437251412566. Online ahead of print. doi: 10.1177/07067437251412566 (PMC12890602; doi:10.1177/07067437251412566)
Supplement: sj-docx-2-cpa-10.1177_07067437251412566 - Supplemental material for Unacceptable Experiences Reported by Undergraduate Students and Their Associations With Mental Health, Well-Being and Academic Performance: U-Flourish Student Well-Being Research: Expériences inacceptables signalées par les étudiant [file sj-docx-2-cpa-10.1177_07067437251412566.docx]

| **Supplementary Table 1B**. Results of multivariable log-binomial regression analyses examining associations between unacceptable experiences (Yes, No, or Not Sure)) reported over the academic year and low well-being and past 6-month suicidal thoughts and behaviours reported at the end of the academic year | | | | | | | | | | | | | |
| --- | --- | --- | --- | --- | --- | --- | --- | --- | --- | --- | --- | --- | --- |
|  | | **Low Well-being (SWEMWBS ≤19)** | | | | | | **Suicidal Thoughts & Behaviours (past 6 months)** | | | | | |
|  |  |  |  | **Model 1*** | | **Model 2**** | |  |  | **Model 1*** | | **Model 2**** | |
|  |  | **nTot** | **%Yes** | **RR** | **(95% CI)** | **RR** | **(95% CI)** | **nTot** | **%Yes** | **RR** | **(95% CI)** | **RR** | **(95% CI)** |
| **Sexual Violence or Harassment** | | | |  |  |  |  |  |  |  |  | *Current (past 6 month) suicidal thoughts and behaviours not available on all versions of the Fall baseline survey* | |
|  | No | 2012 | 25.6 | 1.00 | ref | 1.00 | ref | 2289 | 14.9 | 1.00 | ref |  |  |
|  | Yes | 241 | 36.5 | 1.22 | (0.99-1.50) | 1.14 | (0.89-1.20) | 274 | 31.4 | 1.62 | (1.32-1.99) |  |  |
|  | Not Sure | 112 | 33.0 | 0.99 | (0.72-1.38) | 0.98 | (0.74-1.30) | 127 | 38.6 | 1.93 | (1.51-2.47) |  |  |
| **Discrimination** | |  |  |  |  |  |  |  |  |  |  |  |  |
|  | No | 2017 | 25.2 | 1.00 | ref | 1.00 | ref | 2298 | 15.6 | 1.00 | ref |  |  |
|  | Yes | 195 | 41.0 | 1.50 | (1.24-1.80) | 1.43 | (1.21-1.68) | 221 | 32.1 | 1.75 | (1.42-2.16) |  |  |
|  | Not Sure | 154 | 33.8 | 1.21 | (0.98-1.51) | 1.23 | (0.99-1.53) | 170 | 27.7 | 1.50 | (1.16-1.93) |  |  |
| **Bullying/Harassment** | | |  |  |  |  |  |  |  |  |  |  |  |
|  | No | 2104 | 24.8 | 1.00 | ref | 1.00 | ref | 2399 | 15.8 | 1.00 | ref |  |  |
|  | Yes | 174 | 49.4 | 1.68 | (1.41-2.00) | 1.52 | (1.31-1.77) | 194 | 38.1 | 1.77 | (1.42-2.20) |  |  |
|  | Not Sure | 85 | 37.7 | 1.40 | (1.07-1.83) | 1.25 | (0.95-1.65) | 94 | 25.5 | 1.46 | (1.08-1.97) |  |  |
| **Hate Crimes** | |  |  |  |  |  |  |  |  |  |  |  |  |
|  | No | 2250 | 26.4 | 1.00 | ref | 1.00 | ref | 2553 | 17.3 | 1.00 | ref |  |  |
|  | Yes | 64 | 43.8 | 1.37 | (1.01-1.87) | 1.41 | (1.09-1.83) | 78 | 25.6 | 0.99 | (0.66-1.47) |  |  |
|  | Not Sure | 49 | 36.7 | 1.29 | (0.87-1.91) | 1.46 | (1.05-2.05) | 56 | 26.8 | 1.38 | (0.84-2.27) |  |  |
| **Physical Assault** | |  |  |  |  |  |  |  |  |  |  |  |  |
|  | No | 2288 | 26.4 | 1.00 | ref | 1.00 | ref | 2600 | 17.0 | 1.00 | ref |  |  |
|  | Yes | 45 | 57.8 | 1.64 | (1.19-2.24) | 1.57 | (1.18-2.07) | 53 | 41.5 | 1.45 | (0.93-2.27) |  |  |
|  | Not Sure | 31 | 35.5 | 1.18 | (0.72-1.94) | 1.24 | (0.87-1.76) | 35 | 28.6 | 1.80 | (1.06-3.08) |  |  |
| **Model 1 Adjusted for age, gender, lifetime history of mental illness, ethnicity, and parental education level*  ***Model 2 Adjusted for age, gender, lifetime history of mental illness, ethnicity, parental education level, and mental health status at baseline* | | | | | | | | | | | | | |
