# Supplementary material for: Unacceptable Experiences Reported by Undergraduate Students and Their Associations With Mental Health, Well-Being and Academic Performance: U-Flourish Student Well-Being Research: Expériences inacceptables signalées par les étudiants de premier cycle et leurs liens avec la santé mentale, le bien-être et le rendement académique : Programme de recherche U-Flourish sur le bien-être des étudiants
Source: Can J Psychiatry. 2026 Feb 10:07067437251412566. Online ahead of print. doi: 10.1177/07067437251412566 (PMC12890602; doi:10.1177/07067437251412566)
Supplement: sj-docx-3-cpa-10.1177_07067437251412566 - Supplemental material for Unacceptable Experiences Reported by Undergraduate Students and Their Associations With Mental Health, Well-Being and Academic Performance: U-Flourish Student Well-Being Research: Expériences inacceptables signalées par les étudiant [file sj-docx-3-cpa-10.1177_07067437251412566.docx]

| **Supplementary Table 2**. Results of multivariable linear regression examining associations between unacceptable experiences (Yes, No, or Not Sure) and academic performance over the year (Cumulative GPA) | | | | | | | | | | | | | | |
| --- | --- | --- | --- | --- | --- | --- | --- | --- | --- | --- | --- | --- | --- | --- |
|  | | **All Students** | | | | | |  | **First Year Students** | | | | | |
|  | | **n** | **GPA** | |  | | |  | **n** | **GPA** | |  | | |
|  |  |  | **Mean** | **(SD)** | **β** | **(95% CI)** | **p** |  |  | **M** | **(SD)** | **β** | **(95% CI)** | **p** |
| **Sexual Violence or Harassment** | | | |  |  |  |  |  |  |  |  |  |  |  |
|  | No | 2194 | 3.44 | (0.84) | ref | / |  |  | 882 | 3.41 | (0.77) | ref | / |  |
|  | Yes | 270 | 3.33 | (0.80) | -0.05 | (-0.16, 0.05) | 0.30 |  | 119 | 3.19 | (0.82) | -0.11 | (-0.26, 0.04) | 0.15 |
|  | Not Sure | 126 | 3.32 | (0.85) | -0.10 | (-0.24, 0.04) | 0.16 |  | 57 | 3.22 | (0.89) | -0.14 | (-0.34, 0.07) | 0.19 |
| **Discrimination** | |  |  |  |  |  |  |  |  |  |  |  |  |  |
|  | No | 2210 | 3.42 | (0.82) | ref | / |  |  | 897 | 3.39 | (0.75) | ref | / |  |
|  | Yes | 216 | 3.37 | (0.93) | 0.01 | (-0.11, 0.12) | 0.91 |  | 91 | 3.23 | (1.04) | -0.10 | (-0.27, 0.06) | 0.22 |
|  | Not Sure | 162 | 3.34 | (0.95) | -0.04 | (-0.17,0.08) | 0.49 |  | 71 | 3.30 | (0.86) | -0.04 | (-0.22, 0.15) | 0.71 |
| **Bullying/Harassment** | | |  |  |  |  |  |  |  |  |  |  |  |  |
|  | No | 2304 | 3.44 | (0.82) | ref | / |  |  | 926 | 3.40 | (0.76) | ref | / |  |
|  | Yes | 189 | 3.29 | (0.88) | -0.09 | (-0.20, 0.03) | 0.15 |  | 83 | 3.23 | (0.87) | -0.05 | (-0.22, 0.12) | 0.54 |
|  | Not Sure | 93 | 3.24 | (0.93) | -0.18 | (-0.34, -0.01) | 0.04 |  | 49 | 3.09 | (1.07) | -0.29 | (-0.50, -0.07) | 0.01 |
| **Hate Crime** | |  |  |  |  |  |  |  |  |  |  |  |  |  |
|  | No | 2459 | 3.43 | (0.83) | ref | / |  |  | 1000 | 3.39 | (0.77) | ref | / |  |
|  | Yes | 73 | 3.29 | (0.92) | -0.11 | (-0.29, 0.08) | 0.25 |  | 31 | 2.91 | (1.11) | -0.38 | (-0.65, -0.11) | 0.01 |
|  | Not Sure | 54 | 3.27 | (1.03) | -0.12 | (-0.33,0.10) | 0.28 |  | 25 | 3.29 | (0.86) | -0.08 | (-0.38, 0.21) | 0.59 |
| **Physical Assault** | |  |  |  |  |  |  |  |  |  |  |  |  |  |
|  | No | 2500 | 3.42 | (0.84) | ref | / |  |  | 1018 | 3.38 | (0.79) | ref | / |  |
|  | Yes | 52 | 3.31 | (0.71) | -0.08 | (-0.30, 0.14) | 0.47 |  | 25 | 3.26 | (0.70) | -0.11 | (-0.41, 0.19) | 0.47 |
|  | Not Sure | 35 | 3.48 | (0.76) | 0.04 | (-0.22,0.30) | 0.75 |  | 14 | 3.17 | (1.01) | -0.24 | (-0.63, 0.15) | 0.22 |
| *Note: Models adjusted for age, gender, lifetime history of mental illness, parental education, ethnicity, and program of study* | | | | | | | | | | | | | | |
